# Autologous osteochondral graft as treatment for gouty tophus in the talus: A case report

**DOI:** 10.1097/MD.0000000000022537

**Published:** 2021-02-05

**Authors:** Sheng Mei, Xin Zheng, Jingsong Kong, Yang Huang, Chen Tao

**Affiliations:** a Orthopaedics Center, Taizhou Municipal Hospital, Taizhou; b Department of Orthopedics, Tongde Hospital of Zhejiang Province, Hangzhou, Zhejiang Province, China.

**Keywords:** ankle joint, gouty tophus, osteochondral transplantation

## Abstract

**Rationale:**

Gout can cause redness, swelling, local heat, severe pain, and limitation of function of the affected joints and surrounding tissues. Gouty tophi are commonly found in the auricle, joints, Achilles tendon and tarsal bursa. However, gouty tophi rarely affect the talus.

**Patient concerns:**

We report a case of a 35-year-old man with a history of a sprained left ankle (six years before presentation), who presented with atraumatic and progressive pain, which the patient has been experiencing for a year.

**Diagnosis:**

The patient was diagnosed with ankle pain with a gouty stone in the talus.

**Interventions:**

The patient was treated with autologous osteochondral transplantation.

**Outcomes:**

During the two-year follow-up period, the patient's ankle joint underwent functional recovery and pain relief. Furthermore, the patient's Baird-Jackson ankle score improved from 80 to 95.

**Lessons:**

The gold standard for the diagnosis of gouty tophus in the talus is intraoperative arthroscopy and pathology. The presented case achieved satisfactory clinical effects with autologous osteochondral transplantation as the treatment for gouty tophus in the talus, and obtained an ideal hyaline cartilage repair with restored ankle joint function.

## 1 Introduction

Subcutaneous gouty tophi and chronic gouty arthritis are caused by a long-term history of hyperuricemia, which results in the deposition of monosodium urate monohydrate (MSU) crystals in the skin, synovium tissues in the joint, cartilage, and bone, and the surrounding soft tissues.^[1]^ Furthermore, gout can cause redness, swelling, local heat, severe pain, and a limitation of function of the affected joints and surrounding tissues. A number of complex interacting processes are responsible for the pathophysiology of gout: metabolic, genetic, and other factors that result in hyperuricemia; physiologic, metabolic, and other characteristics responsible for crystal formation; acute inflammatory processes and the involvement of cellular and soluble inflammatory and innate immune processes. In addition, these chronic inflammatory processes and effects of crystals and immune cells on osteoclasts, osteoblasts, and chondrocytes contribute to the formation of tophi, and bone erosion, cartilage attrition and joint injury.

Gouty tophi are commonly found in the auricle, joints, Achilles tendon, and tarsal bursa.^[2]^ However, gouty tophi rarely affect the talus. There are merely few case reports of gouty tophi related to the talus. The mainstay treatment of a talus tophus is surgery, including arthroscopic debridement, simple curettage, cement filling, and microfracture and autologous or allograft osteochondral transplantation. Among these techniques, arthroscopic debridement,^[3]^ simple curettage,^[4]^ or cement filling^[5]^ can relieve symptoms for a certain period of time, but cannot repair the damaged cartilage. The microfracture technique allows the bone marrow to flow out through the subchondral bone by drilling, allowing mesenchymal stem cells in the bone marrow to expand locally, forming new cartilage. This is a suitable treatment for cartilage damage in small-areas. However, the long-term effect of the newly formed fibrocartilage remains poor.^[6]^ In the present study, we report a rare case of gouty tophus in the talus that was successfully treated through autologous osteochondral transplantation.

## 2 Case presentation

**Figure 1:**
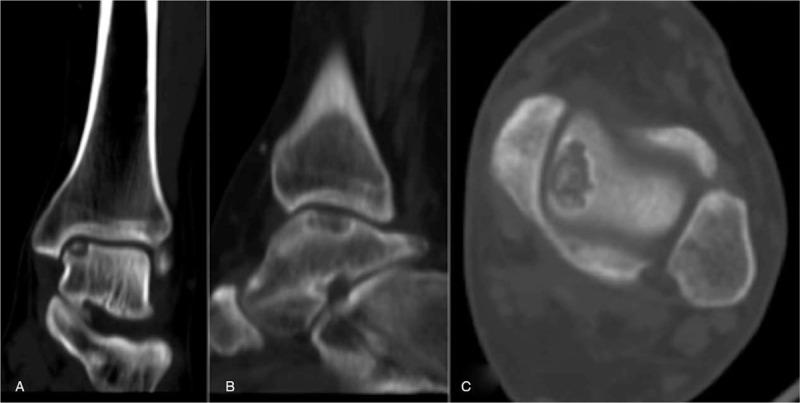
The preoperative computed tomography (CT) images of the left ankle joint present the 15.09 × 9.54 × 5.72 mm low density cystic defect area in the talus from the coronal (A), sagittal (B), and transactional (C) views.

**Figure 2:**
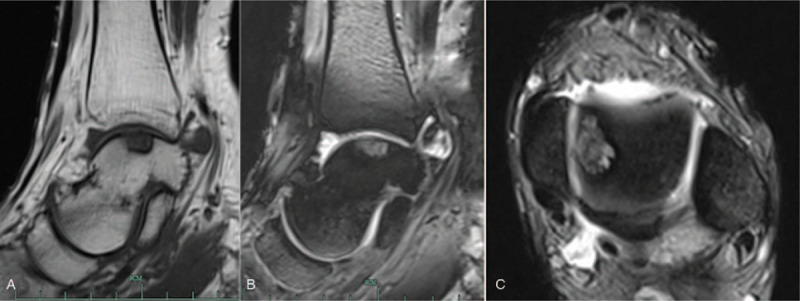
The preoperative magnetic resonance imaging (MRI) of the left ankle is shown, as well as the high signal intensity on the sagittal T1 weighted imaging, with low signal strength (A), sagittal (B), and transversal (C) T2 weighted imaging.

The patient was a 35-year-old man with a history of a sprained left ankle (6 years before presentation), who presented with atraumatic and progressive pain, which the patient has been experiencing for a year. The patient experienced ankle pain during daily activities, and the patient's walking and running abilities were moderately limited. Furthermore, although the patient could perform normal labor, the patient could not perform intensive labor work. The patient had no previous medical history of hypertension, diabetes, gout, or other family history of genetic diseases. The physical examination revealed local swelling of the left ankle joint and lateral tenderness, but there was no significant abnormality in ankle joint activity. According to the Baird-Jackson ankle score,^[7]^ the patient's score was 80. The routine blood test revealed that the level of inflammation cytokines was within the normal range. However, the serum uric acid level was 626 μmol/L (normal: 149–416 μmol/L). The computed tomography (CT) imaging of the left tarsal joint revealed a low-density cystic structure in the talar, which measured at 15.09 × 9.54 × 5.72 mm (Fig. 1). The magnetic resonance imaging (MRI) of the left ankle revealed low signal intensity on the T1-weighted images and an elliptic shaped lesion with high signal intensity on the T2-weighted images (Fig. 2). The initial diagnosis was osteochondritis dissecans of the talus.

**Figure 3:**
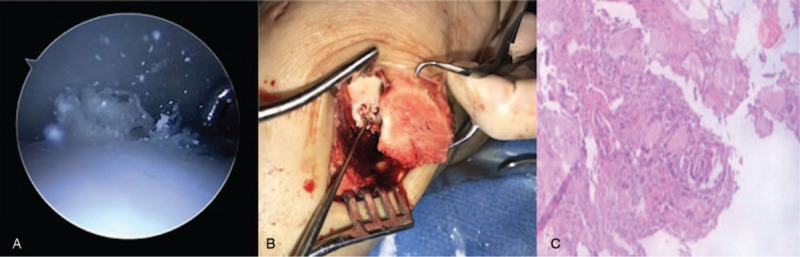
The intraoperative arthroscopic images present the crystals (indicated by the red arrows) (A). The surgical exploration confirmed the presence of the monosodium urate crystal deposition in the medial cartilage of the talus under the cartilage (B). The hematoxylin and eosin histological analysis of the crystalloid materials is shown (C).

**Figure 4:**
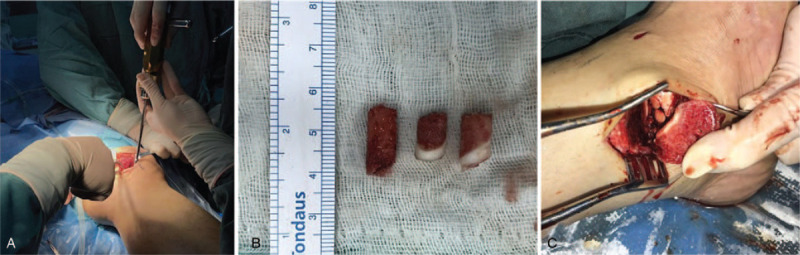
The left femoral condyle cartilage was harvested (A and B) and transplanted to the talus defect (C).

**Figure 5:**
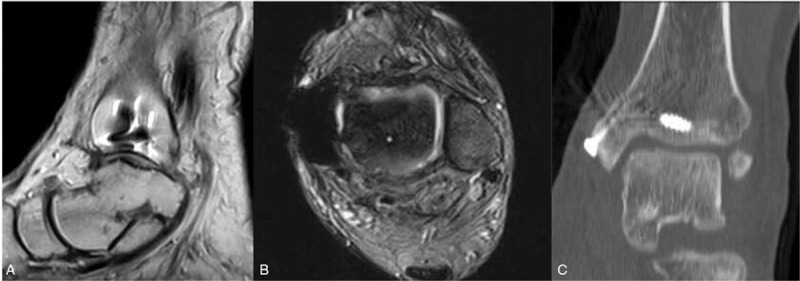
The magnetic resonance (MR) and computed tomography (CT) images of the left ankle joint after 2 years, post-surgery. The sagittal T1-weighted (A) and transverse T2-weighted (B) MRI are shown. The CT image of the left ankle joint is shown (C).

In order to surgically explore the joint, the patient was placed in the supine position. Under general anesthesia, the left ankle joint cavity was probed by laparoscopy. Synovial hyperplasia was evident in the joint cavity, and crystalloid materials were observed to be floating in this region (Fig. 3A). A 7-cm skin incision was made along the lateral left ankle joint. The deep fascia of the skin was cut open to the bone. An osteotomy was performed with a jigsaw to expose the medial condyle of the talus. The local cartilage on the medial side wall of the talus was collapsed, and a significant amount of MSU crystal deposition was observed to be disassociated from the cartilage (Fig. 3B). Furthermore, the crystalloid material was completely scraped off, and the size of the talar cartilage defect was approximately 1.5 × 1.1 cm. In order to maintaining joint stability, the application of the graft in gouty tophus of the talus is quite significant. An 8 cm incision was made along the medial side of the left knee. A deep fascia of the subcutaneous tissue was cut through until the femoral bone was reached. The left femoral condyle cartilage was harvested, as shown in Fig. 4A and B. The cartilage of the medial femur was harvested and transplanted into the talus defect (Fig. 4C). The intraoperative histological examination of the crystalloid material suggested that the eosinophilic structures were surrounded by a large number of macrophages (Fig. 3C). Hence, the diagnosis of gouty tophus was confirmed. In addition, the patient had to take medication to speed recovery. The level of urinary acid rapidly decreased after the treatment, and remained within the normal range (355 μmol/L). During the 2-year postoperation follow-up period, the patient received regular monthly check-ups at the clinic. The patient had mild ankle pain when performing a large range of movement, but there was no pain during walking, and no limping or mild pain during running occurred. The patient could perform the usual activities, and the Baird-Jackon score increased to 95, which was significantly better, when compared with that before the surgery. The CT and MRI of the left ankle joint suggested that the majority of the talus defect was treated (Fig. 5).

## 3 Discussion

In the present case report, a case of gouty tophus in the talus was successfully treated using an autologous osteochondral graft. The patient achieved significant improvement in pain relief and the Baird-Jackson ankle score.

Gout is a crystal-associated arthropathy caused by the deposition of MSU. Imaging examination is critical for the diagnosis of gout. In MRI images, gouty tophi are indicated by low signal densities on T1 weighted imaging (T1WI), and high signal densities with an eccentric round or oval shape and a clear border on T2 weighted imaging (T2WI).^[5]^ However, the differential diagnosis of gouty tophus from exfoliative osteochondritis and bone tumors can be difficult due to the similarities in features revealed through imaging, and its diverse clinical manifestations. For cases with imaging results that indicate that the gout is combined with hyperuricemia, gouty tophus should be considered as the first diagnosis. The intraoperative arthroscopic exploration and pathological analysis further confirmed the diagnosis as the gold standard.

Allogeneic osteochondral transplantation harvests the osteochondral cartilage of a size that matches the size of the defect, and offers more choices in matching the cartilage tissue size, while avoiding secondary injury at the donor site. However, this technique has some issues, such as immune rejection, disease transmission, and transplantation material absorption.^[8]^ Autologous osteochondral transplantation involves the transplantation of a healthy plug that contains articular cartilage, cartilage tidemark, and subchondral bone to the lesion area that matches the size of the lesion area. The advantage of this technique is the use of a hyaline cartilage to repair the defect, and maintain the height and shape of the joint.^[9]^ Compared with simple curettage, cement filling and other methods, osteochondral grafting can simultaneously fill the cancellous bone, restore the cartilage surface to support the biomechanical properties of the composite talus, and restore the biomechanical properties of the original hyaline cartilage for activities. This technique is a better choice for patients who require high activity in the area. Due to the difference in cartilage thicknesses, the height of the bone plane (ankle, 1.0–1.66 mm; knee, 1.69–2.25 mm) in the left ankle CT images may appear to be different (Fig. 4C).^[10,11]^ However, the actual cartilage surface is even. Thus, there will be no adverse effect on the ankle.

Autologous osteochondral transplantation also has disadvantages. This procedure is generally suitable for localized cartilage defects with a defect area of ≤2.5 cm^2^. If the defect area is large, there will inevitably be dead space between the multiple cylinder grafts, which will eventually be filled with fibrous tissue. In addition, the source of autologous cartilage is limited when the damaged area is too big.^[12]^

Since it is rare for gouty tophi to affect the talus, and that this is a single case report, this successful experience could be useful for doctors for future reference. The main limitation of the present study is that the follow-up period was relatively short.

## Author contributions

**Conceptualization:** Tao Chen, Jingsong Kong.

**Data curation:** Tao Chen, Jingsong Kong, Yang Huang.

**Methodology:** Xin Zheng.

**Writing – original draft:** Sheng Mei, Tao Chen, Yang Huang.

**Writing – review & editing:** Sheng Mei, Xin Zheng.
